# Neurodevelopment as an alternative to neuroprogression to explain cognitive functioning in bipolar disorder

**DOI:** 10.1017/S0033291724003210

**Published:** 2024-12

**Authors:** Diego J. Martino

**Affiliations:** 1 National Council of Scientific and Technical Research (CONICET), Buenos Aires, Argentina; 2 Institute of Cognitive and Translational Neuroscience (INCyT), INECO Foundation, Favaloro University, Buenos Aires, Argentina

**Keywords:** bipolar disorder, cognitive impairmets, neurocognition, neurodevelopment, neuroprogression

## Introduction

Over the past 25 years, a growing body of evidence has emerged regarding the presence of cognitive deficits in bipolar disorder (BD) even after remission of mood symptoms. Several meta-analyses consistently reported that euthymic patients as a group show, despite preserved IQ, deficits in verbal memory, attention and executive functions with medium to large effect sizes (Arts, Jabben, Krabbendam, & van Os, 2008; Bora, Yucel, & Pantelis, 2009; Mann-Wrobel, Carreno, & Dickinson, 2011; Torres, Boudreau, & Yatham, 2007. The same profile has been described for both BD type I and BD type II (Bora, Yücel, Pantelis, & Berk, 2011; Dickinson, Becerra, & Coombes, 2017). The association between cognitive impairments and difficulties in psychosocial functioning was also systematically replicated in both cross-sectional and longitudinal studies (Depp et al., 2012; Lomastro, Valerio, Szmulewicz, & Martino, 2021).

In a further step, research advanced by demonstrating the heterogeneity in cognitive profiles within the patient group. The early studies established different cut-off points to define cognitive deficits; depending on whether they were more lax (e.g. performance below 2 standard deviations in a test) or more stringent (e.g. performance below 2 standard deviations on 2 different cognitive domain tests), between one and two thirds of euthymic patients were reported to have no significant cognitive impairment (Gualtieri & Morgan, 2008; Iverson, Brooks, Langenecker, & Young, 2011; Martino et al., 2008; Reichenberg et al., 2009). One study used simultaneously ‘soft’ and ‘hard’ cut-off points to define cognitive impairment. This study reported that while 30% of the sample of euthymic BD patients had cognitive and psychosocial functioning indistinguishable from that of healthy controls, another 30% of patients had cognitive impairment more profound and extensive than that usually reported in the literature (Martino et al., 2014). A series of subsequent studies used multivariate statistics (i.e. cluster analysis or latent class analysis) instead of cut-off points to explore cognitive heterogeneity. The results were also consistent, with about one-third of patients showing intact neurocognitive functioning, one-third selective deficits, and one-third global impairment (Burdick et al., 2014; Lewandowski, Sperry, Cohen, & Ongür, 2014; Solé et al., 2016; Volkert et al., 2015).

Despite consistent advances in this field, there is still controversy regarding how to explain the heterogeneity of cognitive deficits, as well as their origin and trajectory throughout the course of the illness (Samamé, 2024; Vieta, 2024). Two hypotheses have been put forward as alternatives to help explain these issues, neuroprogression and neurodevelopmental abnormalities as detailed below.

## Neuroprogression as determinant of cognitive impairment

Neuroprogression was conceptualized as a pathological rewiring of the brain that takes place in parallel with the functioning and clinical deterioration in the course of BD (Berk, 2009; Kapczinski et al., 2008). The proponents of this hypothesis argue that BD has a progressive clinical course, evolving through different stages from at-risk to more severe and disabling presentations (Berk et al., 2007, 2017; Gama, Kunz, Magalhães, & Kapczinski, 2013; Kapczinski et al., 2009). Regarding neurocognition, this hypothesis proposes a progression from no cognitive impairments in the premorbid and early stages of illness, in which periods of euthymia are well defined, to severe cognitive impairments in later stages of the disorder as a consequence of successive mood episodes (Cardoso, Bauer, Meyer, Kapczinski, & Soares, 2015; Kapczinski et al., 2009; Rosa et al., 2014). From this perspective, heterogeneity in cognitive functioning might be explained based on the stage of the illness. However, empirical evidence supporting both premises (i.e. absence of cognitive deficits in early stages and worsening of these throughout the course of the illness) is scarce and limited.

The notion of ‘no cognitive impairments in the premorbid and early stages of illness’ might be supported by some cohort studies showing that, unlike in schizophrenia, the IQ or other measures of general intelligence or academic performance of people who later develop BD are not affected (for a review see Martino, Samamé, Ibañez, & Strejilevich, 2015). This is not surprising, given that as mentioned above, even most studies done with BD patients report premorbid or current IQ values comparable to those of healthy controls (Arts et al., 2008; Bora et al., 2009; Mann-Wrobel et al., 2011; Torres et al., 2007). However, it cannot be inferred from these results that patients with BD do not have cognitive deficits in premorbid or early stages, since they may have impairment of different cognitive domains (i.e. verbal memory, attention, executive function) that are not reflected in IQ deviations. In fact, the few studies evaluating cognitive domains in subjects at risk who later develop BD show worse performance than controls (for a review see Martino et al., 2015). Furthermore, different studies and meta-analyses found that healthy relatives of subjects with BD show some degree of impairments in executive functions, attention and verbal memory (Arts et al., 2008; Bora et al., 2009). It is evidently contradictory to assume that patients with BD do not have cognitive impairment in the premorbid or early stages of the illness but that their healthy relatives do. Finally, another line of evidence that conflicts with this premise of neuroprogression comes from studies conducted on first episodes of BD. Different meta-analyses have reported that the profile of cognitive deficits found in patients with a first manic episodes or after their remission is similar to that reported in patients in the middle course of the illness (Bora & Pantelis, 2015; Lee et al., 2014).

**Figure 1.**
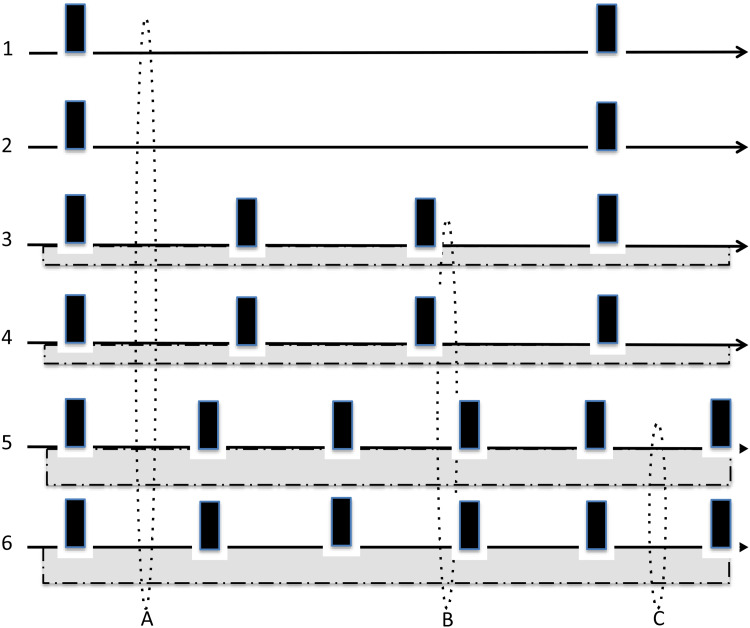
Schematic diagram of the lifetime clinical course of a hypothetical sample of 6 patients with bipolar disorder under the assumption that recurrence of mood episodes (represented by black columns) and cognitive impairment (represented by gray bar with dotted line) is stable from illness onset. The cognitive impairment after a first mood episode is the average of the 6 patients (absent in patients 1–2, moderate in 3–4, and severe in 5–6), but after a third episode it is that of patients 3–6 and after a fifth episode it is that of patients 5–6. Thus comparing cognitive impairment in patients with many *v*. few episodes can lead to the misinterpretation that these features increase with successive episodes even though they were stable. The same positive results would be obtained in correlation analysis between cognitive deficits and number of mood episodes and, therefore, they should not be used as evidence of neuroprogression.

The notion of ‘severe cognitive impairments in later stages of the disorder’ comes from cross-sectional studies showing a positive association between the number of previous mood episodes and the severity of cognitive deficits. This was a finding consistently reported by almost all research groups, either through correlation/regression analyses or by comparing patients with many and few previous mood episodes (López-Jaramillo et al., 2010; Martino, Igoa, Marengo, Scápola, & Strejilevich, 2018a; Robinson & Ferrier, 2006; Torres et al., 2010). The authors proposing the neuroprogression hypothesis interpret these findings as meaning that successive mood episodes lead to progressive cognitive impairment (Berk et al., 2017; Cardoso et al., 2015; Kapczinski et al., 2009). However, it has been noted that the direction of causality of this association cannot be inferred from cross-sectional studies (Martino et al., 2013; Martino, Samamé, Marengo, Igoa, & Strejilevich, 2016). Even if cognitive deficits were stable over the course of the disorder – and related with mood episodes – the same pattern of findings might be observed (Fig. 1). Consequently, this type of results should not be taken as evidence supporting the neuroprogression hypothesis.

On the other hand, further lines of evidence also conflict with the notion of progressive cognitive deficits in BD. For example, results of neurocognitive studies in older adults with BD show similar results to those reported in younger patients, suggesting no progression of impairment (for a review see Samamé, Martino, & Strejilevich, 2013). More importantly, early meta-analyses of longitudinal studies show stability of cognitive deficits throughout the course of the illness (Bora & Özerdem, 2017; Samamé, Martino, & Strejilevich, 2014). Another meta-analysis focused on longitudinal studies of patients with recent onset or older adults and found the same pattern of cognitive stability reported in the middle course of the illness (Szmulewicz, Valerio, & Martino, 2020). Furthermore, a longitudinal study of around 6 years of follow-up specifically designed to measure this association found that although verbal memory deficits and executive dysfunction were associated with the number of (hypo)manic episodes and with the time spent with (hypo)manic symptomatology during follow-up, successive episodes did not modify cognitive performance (Martino et al., 2018a). Finally, more recent longitudinal studies in patients belonging to different age groups and with up to 10 years of follow-up also consistently show stability of cognitive performance throughout the course of the illness (Flaaten et al., 2024; Frías et al., 2017; Jiménez-López et al., 2019; Schouws, Comijs, Dols, Beekman, & Stek, 2016; Sparding et al., 2021). The same stability profile resulted from a recent meta-analysis based exclusively on controlled studies of long-term (>5 years) cognitive outcomes, with a weighted mean follow-up period of 8.9 years (Samamé, Cattaneo, Richaud, Strejilevich, & Aprahamian, 2022).

Overall, empirical evidence does not support any of the premises of the neuroprogression hypothesis. In this context, another alternative hypothesis proposes neurodevelopmental abnormalities in a subset of BD patients to explain the heterogeneity of cognitive deficits, their origin and trajectory throughout the course of the illness.

## Neurodevelopment as determinant of cognitive impairment

As mentioned above, one-third of BD patients have global cognitive impairment, and converging evidence from studies of first episodes, at-risk individuals who later develop BD, and healthy family members suggests that these deficits would precede the first mood episodes. Conversely, another third of patients have intact neurocognitive functioning even after the onset of the disease. Interestingly, some recent studies have shown that a similar pattern of cognitive heterogeneity reported in patients is found in their healthy relatives (Bora et al., 2019; Russo et al., 2017; Valli et al., 2021). Thus, the neurocognitive impairment reported in healthy relatives would depend fundamentally on those of patients with global cognitive deficits, while the same pattern of indemnity would be reproduced in relatives of patients with intact neurocognitive functioning. These same profiles of cognitive subtypes have shown no change over time in longitudinal analyses and have been reproduced in older adults with BD, suggesting the stability of these throughout the course of the illness (Martino, Marengo, Igoa, & Strejilevich, 2018b; Martino, Scápola, & Strejilevich, 2017), which is also consistent with the results of longitudinal studies on neurocognition (Bora & Özerdem, 2017; Samamé et al., 2014, 2022; Szmulewicz et al., 2020). Taken together, these findings support the hypothesis of neurodevelopmental abnormalities. This hypothesis proposes abnormalities in brain development (either genetic or environmental in origin) might affect a subset of BD patients and play a relevant role in the development of cognitive deficits, premorbid behavioral disorders or neurological symptoms.

Other lines of evidence also support this hypothesis. For example, as mentioned above, studies often report average academic performance or general intelligence similar to controls in subjects who later develop BD. However, two of the largest cohort studies reported a bimodal pattern in which both high and low performance increased the risk of BD suggesting some underlying pathophysiological differences in subsets of subjects who later develop the illness (Gale et al., 2013; MacCabe et al., 2010). Some prospective cohort studies in at-risk subjects and youth with BD are also consistent with the neurodevelopmental hypothesis. Thus, it has been reported that while some offspring of patients with BD develop the illness without major premorbid abnormalities, others may show cognitive or socialization problems, schizoid or attention-deficit hyperactivity disorder (ADHD) before the onset (Duffy et al., 2014). Similarly, another study in youth with BD reported that those with low neurocognitive functioning had a higher prevalence of ADHD, more difficulties in academic and social functioning, and more manic and depressive symptoms over 2.5 years of follow-up (Frías et al., 2017). These results are consistent with those observed in adults in the middle course of BD, in which patients with cognitive impairment are those with a high density of mood episodes and poor psychosocial functioning, whereas patients with intact cognitive functioning tend to have a more benign clinical course and better functional outcome (Flaaten et al., 2024; Lomastro et al., 2021; Martino et al., 2013; 2016; Reinares et al., 2013; Valerio, Lomastro, & Martino, 2020). Thus, even when cognitive deficits were stable throughout the course of the illness, they would be more severe in subjects with a greater density of mood episodes (Fig. 1) (Martino et al., 2013, 2016). The cause of this association is currently a matter of speculation and may be the focus of future research. It could be hypothesized that both features have a common cause depending on damage to structures (e.g. corticostriatal pathways) involved in both mood regulation and neurocognitive functioning. Alternatively, it could be speculated that those patients with global cognitive deficits might have a worse clinical course (i.e. greater risk of recurrences) as a consequence of their lower adherence to medication or to their lower ability to recognize prodromal symptoms, to maintaining regular lifestyles, or to obtain benefits from psychosocial approaches such as psychoeducation.

Finally, another line of evidence consistent with the model of neurodevelopmental abnormalities in BD comes from studies of soft neurological signs (NSS), which refer to subtle impairments in sensory integration, motor coordination, and the sequencing of complex motor acts. These neurological dysfunctions linked to developmental abnormalities are more prevalent in BD than in healthy subjects, and have been associated with lower premorbid IQ and severe cognitive impairment (Baş, Poyraz, Baş, Poyraz, & Tosun, 2015; Bora, Akgül, Ceylan, & Özerdem, 2018; Valerio, Lomastro, Igoa, & Martino, 2023).

## Discussion and future directions

Much progress has been made in understanding neurocognitive functioning in BD, although it is still difficult to obtain a comprehensive view of the topic. Neuroprogression was proposed as a hypothesis that could contribute to explaining heterogeneity, origins, and trajectory of cognitive deficits in BD, although its premises (i.e. absence of cognitive deficits in early stages and progressive cognitive decline with successive episodes) are not supported by empirical data.

**Figure 2.**
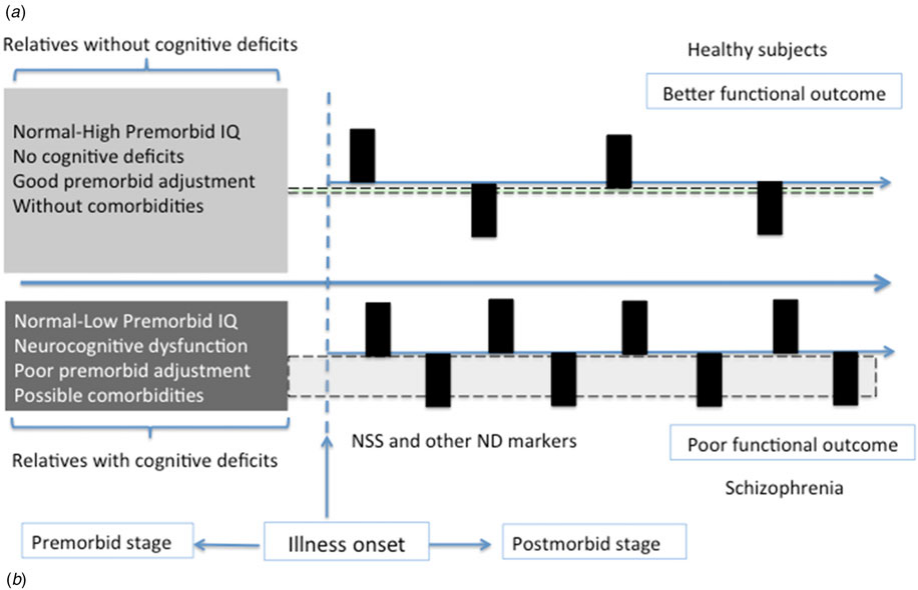
Schematic profiles of bipolar patients with and without neurodevelopmental abnormalities. (*a*) Patients without neurodevelopmental abnormalities do not have premorbid disturbances or neurocognitive dysfunction (gray bar with dotted line) before or after the onset of the illness. In the postmorbid stage, they have a more benign clinical course with a lower density of mood episodes (black columns) and better functional outcome similar – in some cases – to healthy subjects. (*b*) Patients with neurodevelopmental abnormalities have different premorbid disturbances such as socialization and academic problems or comorbidities (e.g. schizoid or ADHD). Even though they have normal-low IQ, they may show impairment in measures of verbal memory, attention or executive functions (gray bar with dotted line) in the premorbid stage that are maintained after the illness onset. In the postmorbid stage, they have a higher density of mood episodes (black columns) and worse functional outcome similar – in some cases – to subjects with schizophrenia.

The alternative hypothesis of neurodevelopmental abnormalities seems to harmonize better with empirical data available to date (Fig. 2). According to this proposal, the global cognitive dysfunction observed in one-third of BD patients as impairments in verbal memory, attention, and executive functions would be present prior to the illness onset (as suggested by the results of studies of subjects at risk who later develop BD, those in their first episode, and those of healthy relatives). Presumably, these would be patients with a normal-low premorbid IQ, and with a higher risk of academic and social maladjustments and a higher prevalence of comorbidities (i.e. schizoid or ADHD) in the premorbid stage. After the onset of BD, these patients would have a higher density of mood episodes, particularly manic ones, throughout their illness. Likewise, this subset of patients would have a higher prevalence of NSS or other neurodevelopmental markers. Both as a consequence of global cognitive impairments and of the worse clinical course, these patients would be those with the poor functional outcome.

In contrast, the other two-thirds of patients would include those with preserved cognitive functioning or selective deficits (Fig. 2). Most of these patients would have normal-high premorbid cognitive functioning, without major social-academic maladjustment or comorbidities before the onset of BD. Consequently, healthy relatives of these patients would not show significant cognitive impairment. After the illness onset, these patients would have a more benign clinical course with lower mood episode density and better psychosocial functioning. Of course, despite this general pattern, there would be high variability regarding clinical course, neurocognitive performance, and functional outcome within this subset of patients. In fact, about half of these patients might have selective cognitive deficits. Selective cognitive deficits could be a consequence of minor neurodevelopmental abnormalities or, alternatively, of different factors related to cognitive deficits in BD: psychiatric (e.g. anxiety disorders, substance use disorders) or medical (e.g. hypothyroidism) comorbidities, adverse effects of medication (e.g. antipsychotics or benzodiazepines), or unhealthy lifestyle habits (e.g. sleep disturbances, sedentary lifestyle, etc.).

Overall, the neurodevelopmental hypothesis is consistent with the early/premorbid origin of cognitive deficits in a subset of BD patients and their stability throughout the course of the illness. It also allows us to understand neurocognitive heterogeneity in BD, with some patients having impairment similar to that reported in people with schizophrenia at one extreme, and subjects with extraordinary cognitive and creative abilities at the other. This great heterogeneity and its explanation through the neurodevelopmental hypothesis raises the question of whether or not it is appropriate to include all these patients within the same psychiatric disorder (i.e. BD), which could be the focus of future studies. Of course, it should ideally include longitudinal follow-up of subjects at risk for BD, although these are the most difficult studies to implement. Alternatively, analysis of the pattern of cognitive heterogeneity in first-episode patients and their longitudinal follow-up (both in terms of clinical course and neurocognitive functioning) could also provide interesting information. Although the literature supports a pattern of stability of cognitive deficits throughout the illness, this must be further explored separately in these patient subsets (with and without neurodevelopment markers). Finally, neuroimaging and laboratory biomarkers might differ in patients with and without neurodevelopment abnormalities, which could also be the focus of future studies. Similarly, family aggregation and genetic studies in these subsets of patients could also contribute relevant information.

Neurocognition has been one of the most prolific fields of research in BD during the last decades and we must make an attempt to integrate current knowledge and continue to move forward. There is potential to improve our understanding of the heterogeneous clinical features (and longitudinal course) and pathophysiological substrate of BD through this path.
